# Ethical challenges in mental health research among internally displaced people: ethical theory and research implementation

**DOI:** 10.1186/1472-6939-14-13

**Published:** 2013-03-12

**Authors:** Chesmal Siriwardhana, Anushka Adikari, Kaushalya Jayaweera, Athula Sumathipala

**Affiliations:** 1 Health Services & Population Research Department, Institute of Psychiatry, King’s College London, London, SE5 8AF, UK; 2 Institute for Research & Development, Battaramulla, 10120, Sri Lanka

**Keywords:** Sri Lanka, Bioethics, Ethical challenges, Internally displaced people, Mental health research, Post-research ethics audit, Practical ethics, Developing world

## Abstract

Millions of people undergo displacement in the world. Internally displaced people (IDP) are especially vulnerable as they are not protected by special legislation in contrast to other migrants. Research conducted among IDPs must be correspondingly sensitive in dealing with ethical issues that may arise. Muslim IDPs in Puttalam district in the North-Western province of Sri Lanka were initially displaced from Northern Sri Lanka due to the conflict in 1991. In the backdrop of a study exploring the prevalence of common mental disorders among the IDPs, researchers encountered various ethical challenges. These included inter-related issues of autonomy, non-maleficence, beneficence, confidentiality and informed consent, and how these were tailored in a culture-specific way to a population that has increased vulnerability. This paper analyses how these ethical issues were perceived, detected and managed by the researchers, and the role of ethics review committees in mental health research concerning IDPs. The relevance of guidelines and methodologies in the context of an atypical study population and the benefit versus risk potential of research for IDPs are also discussed. The limitations that were encountered while dealing with ethical challenges during the study are discussed. The concept of post-research ethical conduct audit is suggested to be considered as a potential step to minimize the exploitation of vulnerable populations such as IDPs in mental health research.

## Introduction

Due to natural and man-made disaster situations, many millions of people are displaced from their homes throughout the world. Some are displaced within the borders of their own countries and are categorized as internally displaced people (IDP), while some are displaced outside of country borders. The latter are considered as refugees and are entitled to be protected by laws of the host country, albeit with certain limitations [1]. However, IDPs are usually not protected by any specific laws as they are often displaced and confined due to political or other forms of violent, lawless situations erupting within their countries, thus increasing their vulnerability [1].

The global literature is saturated with research conducted about mental health issues among refugees. Such studies are usually conducted in the immediate or late aftermath of often violent and traumatic displacement ordeals and involve highly traumatized adult and child populations [2,3], and may consist of interventions or epidemiological studies [4,5]. All types of research carry an inherent risk to the participants, especially due to the natural vulnerability of these populations.

Psychiatric/mental health research is conducted and regulated by general bioethical principles [6]. More specifically, human subject research ethics, clinical trial ethics and other forms of ethical guidelines oversee the regulation of mental health research, similar to other forms of biological research [7,8]. However, there are many specific ethical issues related to psychiatric/mental health research conducted among vulnerable populations such as refugees or IDPs [2]. Understandably, these issues stem from the highly traumatic experiences of these populations, along with their cultural and social backgrounds, especially in the developing country setting [2]. Furthermore, lack of knowledge and awareness about proper ethical practices among researchers, essential for conducting studies among vulnerable populations add to the risk of exploitation [1]. Existing regulatory frameworks, ethical guidelines and expertise of ethics review committees may not be sufficient to provide adequate regulation of research among displaced populations [8]. Another factor to be considered is the lack of awareness/education among the participant communities themselves about potentially harmful research, which can effectively increase the risk of exploitation. The combination of participant population characteristics, inexperienced researchers and weak regulatory frameworks stand to increase unethical and exploitative research being conducted, and the negative effects can be especially salient in the context of mental health research among the displaced.

This paper aims to present a mental health study conducted among a displaced population in the developing country setting of Sri Lanka as an example in order to discuss potential ethical challenges that may arise. We will discuss the potential ethical issues perceived by the researchers and ethical committees, and how these problems were planned to be addressed. We will also present the actual ethical challenges that arose while the study was being conducted in the field setting and discuss how they were addressed. The paper will try to explore these issues in the context of the main bioethical principles of autonomy, non-maleficence, beneficence and justice. Concepts of informed consent, confidentiality, risk and potential benefit will be also discussed in the context of the study background. The study being discussed in this paper was approved by the Psychiatry, Nursing and Midwifery Research Ethics Sub Committee, King’s College London (PNM RESC KCL) and the Ethical Review Committee of the Faculty of Medicine, University of Sri Jayewardenepura (ERC FM SJP), Sri Lanka. However, it was deemed unnecessary by the authors to obtain ethical approval to compile this specific manuscript, as it contains only a theoretical exploration of ethical challenges, and does not include any findings from the original study.

### Displacement in Sri Lanka

Sri Lanka is a multi-ethnic society and had a mid-year population of 20 million estimated in 2007 [9]. It has seen many instances of characteristically unpredictable and fluid internal displacement of people in the recent past. The main reasons for mass displacement were the recently concluded civil conflict that raged for three decades and the 2004 Tsunami. Other cyclic natural disasters (floods, cyclones, landslides), rural poverty, economic difficulties and political upheavals also act as push factors for internal displacement.

### Common mental disorders and association with resilience among internally displaced people in the Puttalam district of Sri Lanka (COMRAID) study

During the thirty years of conflict, thousands of people have been internally and externally displaced from the northern, eastern and north central provinces of Sri Lanka [10]. Many of these IDP have undergone psychological trauma and a cross sectional study was proposed to measure the prevalence of common mental disorders among a specific subgroup (COMRAID study). As a separate component, a qualitative study utilizing individual in-depth interviews was also conducted. Reasons for this specific sub-population of IDPs being selected for such a study include unique nature of their displacement and extended time period of over 20 years in displacement.

COMRAID study was conducted among a population of Muslims (considered as a separate ethnic group in Sri Lanka, whose religion is Islam, and speak mainly Tamil language) displaced from their homes in northern Sri Lanka due to the conflict in 1991 and living in displaced camps and settlements in the Puttalam district, a part of the North Western province geographically close to conflict areas [11,12]. This IDP population was scattered across several camps and settlements at the time of the study. These IDPs were mainly farmers and fishermen at the time of displacement while some members of the community were educated up to university level. They lived together with Sinhala and Tamil ethnic groups [13]. However, these characteristics changed during the post-displacement period. Although a part of the community managed to continue their livelihoods in fishing, the farmers were reduced to day-wage manual laborers in the new resettlement areas. Education was disrupted for those who were displaced as children. Their community became increasingly inter-dependent, closed to outsiders, especially in the backdrop of increasing hostility from the host community [14]. The community grew in numbers, with several generations being born in displacement camps and settlements, creating inter-generation cultural issues as well as socio-economic issues such as unemployment, low education and lack of resources [15]. At the time of the COMRAID study, the three-decade conflict had just concluded, presenting the IDPs with the possibility of returning to areas of origin. However, return migration was not considered as an option by majority of the IDPs, as the areas of origin were completely devoid of resources, lacking in health, education and other infrastructure support systems.

### Practical ethics – perceptions of ethical requirements

In this section of the paper, we present information about the ethical review process that the COMRAID study went through before being conducted. This process involved two main stages; initial funding grant application ethics component and the application for ethical approval to start data collection.

In the first stage, as the COMRAID study was funded as a part of a fellowship grant, the initial grant application contained a section on ethics. This section prompted the investigator to perceive, analyze and list potential ethical issues that may be related to the study design. The grant applicant had to present ways of addressing these perceived ethical issues. Reviewers of the grant application subsequently scrutinized the indicated potential ethical issues and proposed management.

In the second stage, applications were made to two separate ethical review committees; Psychiatry, Nursing and Midwifery research ethics subcommittee, King’s College London (PNM RESC KCL), UK and the Ethics Review Committee of the Faculty of Medicine, University of Sri Jayewardenepura (ERC FM SJP), Sri Lanka. These two applications were made on the basis that the study was being conducted in Sri Lanka and that the principal investigator was attached to King’s College London under the fellowship grant.

### Practical ethics – input from regulatory bodies

Several potential ethical issues perceived by the investigators were presented in the grant application to the funding body. They included issues related to the confidentiality of participant information, difficulties and suitability of eliciting information on past trauma and dealing with participants identified to be suffering from serious mental illnesses. These concerns stemmed from the forced nature of displacement of potential participants, the closed nature of their community, lack of proper communication channels with authorities (government and other), the risk versus benefit dilemma of recapturing past trauma and the non-therapeutic design of the study. Other ethical challenges perceived by the investigators related to the specifics of religion, culture and traditions of the study participants. The mainly Muslim study population was known to be highly traditional, strongly religious and closed against outsiders. Due to these reasons, we were unsure of using non-Muslim researchers from outside communities (field research assistants for the house to house survey). This presented a logistical problem (finding suitable Muslim research assistants) and an ethical issue (is it ethical to use exclusively Muslim research assistants or vice versa). Another ethical issue stemming from the specificity of the Muslim study population was the need to use gender matching research assistants in the study. The community was known to be male dominated, females were known to be segregated and not encouraged for direct interaction with outsiders (especially male outsiders) and not keen on such interaction, let alone share confidential information, thus prompting the need for the use of gender matching research assistants.

The ethical review committee of the Faculty of Medicine, Sri Jayewardenepura University in Sri Lanka approved the study without comments. The PNM RESC of King’s College London raised several important points on the initial application. The committee felt that due to the sensitive nature of the information required and vulnerability of the population, some of the proposed study instruments were inappropriate and that the duration of interviews should be reduced. The initially proposed methodology of using focus groups for the qualitative component was deemed inappropriate by the reviewers, and one-to-one interviews were suggested instead. This was an important suggestion to protect the autonomy of participants and to ensure the confidentiality of information. Assurance of confidentiality of the information obtained from the participants, especially if research assistants were recruited from the locality was raised by the reviewers. Concern was expressed about inducement and pressure to participate, especially in the background of cultural and social contexts of the population. Another important point raised by the committee was the eventuality of discovering suicidal ideations/attempts by a participant and how to address such situations. The ethics committee also emphasized on ensuring the accuracy of diagnosing serious mental illness among participants and proposed measures to deal with such instances.

The perceived ethical challenges and issues raised by the ethics review committees were addressed in the ethical approval stage, in the study design, in the planning stage and also during the conduction of the study in the actual field setting. We present the various aspects of this process categorized according to key areas of ethics and the consent process. However, these aspects overlap at various stages due to their interlinked nature. For example, autonomy, informed consent and confidentiality are interlinked throughout the consent process.

### Practical ethics – implementation of ethical requirements and the process pathways

#### Autonomy

The concept of autonomy in research mainly contains two aspects; the choice of the participant and non-coercion/non-inducement [16]. Autonomy is a key factor in the decision-making process leading up to the participation in any given research, for participants as well as for researchers. However, the concept of autonomy varies in different cultural settings, and markedly so between western and other parts of the world, especially in Asia and Africa [2].

In our study, we perceived issues in assuring the autonomy and decision making capacity of the participants, given their inherent vulnerability of being members of a displaced community. We also perceived that the fast-changing nature of cultural and social background of participants may present problems in ensuring autonomy and obtaining informed consent.

To address the ethical challenges in ensuring autonomy among this population, we were required to assess the characteristics of the IDPs, their experiences of displacement due to conflict, duration of displacement and their relationships with the various stakeholders in charge of serving them; both governmental and non-governmental. Using our knowledge and experience as local (Sri Lankan) researchers, we then took steps to minimize coercion/inducement and maximize autonomy. A previous study on public understanding of research in Sri Lanka provided us with additional insight on how our work may be perceived by potential participants [17].

Our first goal was to introduce ourselves and the purpose of our research to the IDP community, so that our presence among them would not be unexpected and intruding. For this purpose, we approached the District Secretariat (highest civilian administrative body at district level) and the Secretariat for the Northern Displaced Muslims (a dedicated agency for the specific population within the Ministry of Resettlement). Through these agencies, we contacted camp officers in charge of the individual IDP camps. The purpose and methodology of the COMRAID study was explained to them in lay terms and were requested to inform and brief the residents and non-statutory bodies (religious leaders, community leaders) about the study prior to our arrival in camps under their purview. We then approached the religious and community leaders of the selected camps to brief them about the study, and with their consent and acknowledgement, finally approached the IDPs themselves. However, we strictly asked the camp officers and community leaders not to get involved further in the decision making process of the IDPs, and asked them to be neutral. We did not involve them further in the study recruitment process, to prevent intentional or unintentional possibility of coercion/inducement.

The selected IDPs from individual households within the camps were approached by the team of research assistants (composition described later) and established consent procedures were followed. Maximum care was taken to ensure the full range of autonomy, especially among female and younger participants (all participants were above 18 years). A decision was taken by us not to compensate the participants through monetary or any other forms, approved by both ethics review committees, to minimize undue inducement. This is especially important as the IDP communities have previous experiences of being offered incentives for participating in various activities including research, creating a potential risk of developing long term dependency. We approached the participants at their homes and ensured that they did not have to sacrifice their working time to take part in the study. Such steps minimized the need for financial or other forms of compensation.

#### Non-maleficence and beneficence

We tried to limit the potential harm to the participants, as they belong to an already traumatized, highly vulnerable population. An inherent problem in psychiatric research is the recapturing of past trauma without causing re-traumatisation [5]. The recommendations from the ethics committees were valuable in addressing these issues. In the quantitative survey, we removed sections of extreme sensitiveness, which the researchers and one ethics committee both felt were inappropriate to be used among the specific population. These sections included questions on past abuse of sexual nature. A questionnaire on suicidal ideations was removed as suggested by the ethics committee. The duration of the interview process was reduced to one hour, as the ethics committee felt that the original two hour interviews would be harmful due to the sensitive nature of some questions. The team of researchers drew up a step-by-step, regulated action plan in case of a participant being identified as having active suicidal risk or having a serious mental illness. This plan included steps to ensure maximum confidentiality and fast referral to specialist psychiatric care.

In the qualitative study component, we changed the study design to interviews conducted on one-to-one basis based on the comments by the ethics committee, instead of the original design which consisted of holding focus groups. This step was deemed appropriate to minimize potential harm to participants in recounting past trauma among peers while ensuring confidentiality and privacy. We approached a cross section of the IDP community for the qualitative survey.

As a group, the IDPs recruited for COMRAID study had been in displacement for over 20 years without much attention from any relevant authorities. We envisaged that a study such as ours would help to draw attention to numerous issues faced by the IDPs, especially in the domains of health and mental health, which could be beneficial in the long term management of this population. It is an ethical imperative for us as researchers that we disseminate the findings of this study among relevant academic, policy making and stake holder communities in order to make a meaningful contribution to the evidence-based management of IDPs. Findings were also disseminated among the study population in non-academic language, as part of our ethical obligation as researchers.

#### Confidentiality

Various steps were taken to ensure the maximum confidentiality of the information gathered from the participants, including personal information. We incorporated a coding system to the study design, where each participant was assigned a unique code. This code was used by the research assistants at the time of the interview and entered in to the questionnaire booklet. During the data entry process, the unique coding number was used for each participant; no privately identifiable data such as names or addresses were used. Identifiable data was kept secure and separate, accessible only to selected investigators. We followed a similar process in the qualitative component, where separate coding numbers were assigned for the digital recordings of each participant interviewed and kept securely and separately from identifiable data. Due to the closely linked nature of the study population, it was difficult in practice to ensure that the one-to-one interviews were conducted in total privacy, in a setting preferred by the participant. Sometimes, participants wanted their family members to join in to share the recounting of the displacement experience and had to be politely but firmly dissuaded by the investigator. We succeeded in making the participants understand the importance of confidentiality and privacy during the interview for them as well as for the study purposes. It is envisaged that this knowledge will cascade to other members of the IDP community through our participants, and enable the community to protect confidentiality and prevent exploitation from any research they may take part in the future. During both qualitative and quantitative components, normative procedures of data protection were followed for data collection and data storage.

Research assistants were trained to ensure the maximum confidentiality of the information gathered from participants, and this was further enforced by a rigorous supervision process conducted by members of the academic investigator team. The research assistants were chosen from and assigned to work in non-local settings, ensuring confidentiality of information. As described in the section of autonomy, we made sure that the camp officers, community and religious leaders did not have a chance to interfere in the data collection process or try to gain access to the collected data by constantly monitoring the research assistants and their interactions with participants. We also made sure that the medically qualified investigators acted as a buffer between the field workers and community power-groups to minimize disruption and ensure confidentiality of information.

#### Informed consent

The process of informed consent is a crucial ethical aspect of mental health research, especially among vulnerable groups. The dialogue between the researchers and the participant is a key component of the study process, and may (or may not) convey the purpose of the study to potential participants. Lack of successful communication and establishment of a good rapport between the two parties can lead to a breakdown of the research process even before it starts. In addition, the capacity of potential participants to grasp the purpose of the study is crucial to prevent exploitation.

Attempts to address these issues in the study design and implementation stages were made by the research team. People with severe hearing impairment and people who are lacking mental capacity (those suffering from already diagnosed severe mental illness from birth or developed later) were excluded, with the aim of minimizing exploitation.

In the implementation phase, research assistants were intensively trained on ethics involved in research and informed consent. A co-author of this paper, also an investigator of the study, is a World Health Organization (WHO) certified expert in bioethics who has conducted research on informed consent in the Sri Lankan setting [18,19], and his expertise was channeled into the training of research assistants. Extensive role-play was also used in this training. We trained the research assistants on mental health and mental disorders, and identifying mental illness in the community (using a similar method used to train community mental health workers).

At the start of the study, an information leaflet explaining the research project in lay terms was given to each randomly selected participant. Two separate information leaflets were designed for the qualitative and quantitative components. The information leaflets were written in all three official languages of the country (Sinhala, Tamil and English) and participants had the choice of selecting the preferred language version. The research assistants would approach potential participants and explain the study and answer any questions directed by the participants, in a case where they were unable to answer satisfactorily, the participant could directly talk to the principal investigator or any member of the investigator team. The potential participants were asked to take at least a day to make their decision to take part, and if required, to consult the family members and others. Consent forms were printed in the three official languages. Two copies of the consent forms were signed both by the participant and the research assistant, and one copy was given to the participant. Participants could withdraw from the study at any point during the interview process or within a two week period after the date of the interview (this time frame was approved by both ethics committees).

International norms and guidelines on obtaining informed consent were followed in the study. As discussed in other sections, steps were taken to minimize coercion and undue inducement at every stage of the consent process. Medical doctors have a higher standing in Sri Lankan communities and their involvement in the consent process may cause undue inducement in obtaining consent, therefore members of the investigation team refrained from visiting potential participant houses prior to the visit by a research assistant.

#### Other ethical challenges

Apart from the above, several other population-specific ethical challenges were present. As discussed in previous sections, the cultural, religious and social specificities of the majority Muslim study population prompted specific actions to address these ethical issues.

The study had to use gender matching (male participant - male research assistant, female participant – female research assistant) method in conducting the survey. The team of research assistants consisted of five females and one male. Two females and the male were Muslims and the other three females were ethnic Tamil Christians. They were individually selected through newspaper advertisements and a competitive selection process. All selected research assistants could speak the Tamil language, mainly used by the study population. Although the participants were familiar with the IDPs, they were not from the close locality and did not have any links to the IDP community. The gender imbalance among the research assistants constituted to a minor problem as the single male research assistant had to interview most of the male IDPs. However, being a Muslim, he easily established good rapport with the study population and was actually able to conduct interviews with a number of females. The gender imbalance was caused due to the lack of qualified people to work in the geographical area and with the study population.

In the study design, the random selection method of IDP camps/settlements, households from camps and IDPs from households ensured an equal distribution of the sample across the whole IDP community and minimized bias. Selective sampling can be another ethical issue causative to exploitation as researchers can only select participants deemed conducive or positive towards the study [20].

Finally, it must be mentioned that the IDP community warmly welcomed us and gave their fullest support, regardless of poverty or the vulnerable positions they were in. The team of investigators and the field research assistants constantly tried to match this by conducting ethical research to the fullest extent.

## Discussion

In this paper, we explore the ethical challenges associated with mental health research among an internally displaced population. We have taken a mental health study conducted in the Northwestern Sri Lanka among a long-term displaced Muslim population due to civil conflict as an example of identifying and addressing several ethical challenges in such types of research. We have discussed the perceived ethical challenges and how they were addressed in the actual research setting. Finally, we explored the relevance and suitability of the ethical practices followed in our study and how these can be adapted for future mental health research among vulnerable populations.

### Voice of the participant community

By the nature of their displacement experience, these IDPs are a vulnerable community in society. The study population was forced to leave their original homes and belongings, experienced violence during displacement, had to resettle in difficult conditions and had to adapt and survive in sometimes hostile conditions in the host community. Their voices had not been heard by the wider Sri Lankan or international public, although isolated attempts at vocalizing their plight had been made [14,15]. These IDPs did not have a platform to express their views or opinions on various research conducted among them. It would have been ideal to gather ideas and views of IDPs on the types of research they were willing to take part in, and how these studies should be conducted. It would have been also valuable to have a prior understanding of how bioethical concepts such as autonomy, confidentiality and informed consent were perceived. Although we did not have this opportunity before the study, we did manage to gain a deep insight in to the perceptions and needs of the IDP community during the study period, which we hope would act as helpful guidance for future researchers, including ourselves.

#### Vulnerability, autonomy, consent and confidentiality

The IDP community can be seen as easy targets for exploitation, especially for health research. Previous attempts of coercion and inducement by offering compensation in various forms such as temporary supplies of drugs, food, money, or one-off medical testing were reported by our study population when our team approached the IDPs. We had to clearly and effectively communicate our credibility as researchers as well as the nature and purpose of our study to the potential participants, and assure that their vulnerability would not be exploited.

In addition, we were focused on ensuring autonomy of the study participants, especially of women, during the consent process and subsequent interviews. We insisted on obtaining written informed consent and were strict in following existing guidelines on obtaining consent. We had to be sure that the obtained informed consent was genuine and not affected by the vulnerability of the IDPs [2]. We had to pay special attention to power-relations within and outside of the IDP community, especially the power hierarchy related to religious, community and political leaders. We had to minimize their involvement in the decision making process of potential participants by educating the members of the power hierarchy on basic principles of ethics.

Confidentiality was of paramount importance as many of these IDPs had trust issues with many representative bodies of the government and other agencies. We had to overcome this barrier to increase the level of trust placed on the research team to ensure the validity of data. However, we had to achieve this while building up trust and rapport with the IDP community, all the while adhering to strict ethical guidelines on confidentiality.

#### Risk vs benefit

The need for risk vs benefit approach in research, especially qualitative narrative research has been debated in literature [5,21,22]. The risk of re-traumatizing the traumatized opposes the benefits that the IDP community stands to gain through epidemiological, interventional and narrative research. The COMRAID study, being a combination of epidemiological and narrative research is a mix of risks and benefits mentioned. As indicated in the previous sections, we have tried to minimize the harm to participants and expect that the findings will be of benefit to the IDP population. The potential benefits to be shared by the IDP community and the public health sector of Sri Lanka (government and non-government) stem from the belief that that our findings can provide an effective evidence base for policy formulation for IDP management. Other benefits include the narrative retelling of the plight of IDPs, exposing their story to a wider international public. The study aimed for harm minimization through adherence to rigorous ethical standards. However, we do admit that the potential benefits may not be of immediate value to the specific IDP community, but would be more beneficial to the wider IDP communities, both in Sri Lanka and in the global IDP context.

#### Guidelines and methodologies

During the planning, ethical approval-seeking and implementation stages of this study, we encountered an acute lack of awareness about the actual need for mental health research among IDPs and its importance [23]. It should be also noted that apart from the United Nations High Commission for Refugees (UNHCR) Guiding Principles on Internal Displacement, there are no other global guidelines on IDP management [24]. We encountered a lack of guidance on ethical matters as no specific guidelines existed (still do not exist) in Sri Lanka. We had to refer to available international bioethics guidelines, which lacked special emphasis on ethical issues among displaced populations [1]. Although such guidelines are being developed by groups of researchers around the globe, lack of definite guidance can be a cause for unethical research being conducted [1].

Another issue is that the organizations responsible for providing ethical oversight, namely the ethics review committees, lack relevant expertise and knowledge in regulating mental health research among vulnerable groups such as IDPs [2]. This becomes critically important when studies are carried out in different cultural contexts other than those familiar to committee members, especially in developing country settings. Their lack of understanding translates into either unnecessary hindrance to proposed research or recommendation of inappropriate changes in the research population context. We also encountered certain recommendations from ethics committees that were either inappropriate or impractical in the local setting, and informed the committees during such instances.

Ethical challenges were embedded in the COMRAID design itself, related to the survey design of the quantitative component such as numerous questionnaires administered over a long period of time [4]. However, our survey design was deemed optimal for an epidemiological study of this nature. In the COMRAID study we tried to address these issues by cutting out unnecessary, intrusive and long questionnaire components. Interview duration was optimized by rigorous training of the research assistants. As a result, efficiency of the research assistants was increased while reducing participant discomfort.

#### Researcher integrity

It is critically important, though practically difficult that researchers maintain strict personal and professional integrity throughout the research process. Pressures with deliverables and deadlines, accountability to funding agencies, prospective chances of career advancement, personal life pressures and various other factors may influence researchers to take liberties and cut various corners during the research process. Sometimes, certain such decisions can lead to unsound ethical practices, reducing research quality and cause negative impact on the participant community. Although our study team did not consciously or voluntarily compromise research integrity, it can be only proved by a careful post-study ethical audit, conceptually similar to a post study audit of a clinical trial.

#### Lessons for the future

In this paper, we have demonstrated that non-exploitative, ethically sound, methodologically rigorous mental health research can be conducted among vulnerable populations such as IDPs if researchers strictly adhere to ethical guidelines while actively addressing potential ethical issues with the support of ethics review committees. Prior analysis of community perceptions, understanding of research conduct and ethical norms is advocated, in order to give voice to participant community needs. The need for more specific ethical oversight, capacity building among researchers and ethics committees is emphasized as crucial requirements to safe guard the vulnerable populations such as IDPs from harmful research, especially in the field of mental health. The concept of post-research audit of ethical practices is strongly recommended, in order to strengthen existing ethical guidelines and practices.

## Competing interests

CS received a Wellcome Trust Masters Fellowship in Public Health and Tropical Medicine which funded the COMRAID study. CS is currently funded by King’s College London. AS was the Sri Lanka supervisor/sponsor for the grant. AA and KJ were employed as project coordinators by the IRD in the COMRAID study. The work in this paper was not funded by any funding agency.

## Authors’ contributions

CS conceptualized and wrote the paper. AA and KJ contributed their experiences of working as coordinators of the study. AS was a supervisor in the COMRAID study and provided input on ethical issues from the design stage of the study and training on ethics for research assistants. All authors commented on the first draft and subsequent revisions of the paper. All authors read and approved the final manuscript.

## Pre-publication history

The pre-publication history for this paper can be accessed here:

http://www.biomedcentral.com/1472-6939/14/13/prepub
